# Effect of abnormal lipid profile on preeclampsia incidence and severity among Bangladesh women

**DOI:** 10.6026/973206300221617

**Published:** 2026-03-31

**Authors:** Kazi Farhana Begum, Mehera Parveen, Nigar Sultana, Hasna Hena Pervin, Tripti Das, Fahmida Zabin, Humaira Alam

**Affiliations:** 1 Department of Obstetrics and Gynaecology, Bangladesh Medical University, Shahbag, Dhaka, Bangladesh

**Keywords:** Preeclampsia, lipid profile, triglycerides, cholesterol, endothelial dysfunction

## Abstract

Preeclampsia is related to abnormality in lipid profile. Hence, a retrospective cross-sectional study of 300 pregnant women was conducted. Results revealed that 15% developed preeclampsia, including 10% mild and 5% severe cases. Women with preeclampsia had higher pre-gestational BMI and significantly elevated total cholesterol, triglycerides and LDL-C compared to normotensive controls, while HDL-C showed no difference. Severe cases exhibited further increases in cholesterol, triglycerides and LDL-C. Thus, we show the association of abnormal lipid profiles with both the incidence and severity of preeclampsia, supporting their potential role as early predictive markers.

## Background:

Preeclampsia is a pregnancy-specific disorder affecting multiple organ systems and poses significant risks to both maternal and fetal health. It remains a leading contributor to maternal mortality, particularly in low and middle-income countries [1, 2]. Globally, approximately 76,000 women die annually due to preeclampsia and related hypertensive disorders and in regions such as Asia and Africa, nearly 10% of maternal deaths are attributable to these conditions [3]. Overall, hypertensive disorders complicate 5-10% of pregnancies [4]. The prevalence of preeclampsia is estimated at 8-10% of all pregnancies, with higher incidence among women with preexisting hypertension, diabetes mellitus, or a prior history of preeclampsia [5]. The condition is more frequently seen in young, nulliparous women [6]. Additional risk factors include a positive family history of hypertension and there is evidence suggesting a possible link between gestational hypertension and metabolic syndrome [7, 8]. Altered lipid profiles have been proposed as a maternal predisposition factor for preeclampsia, although studies have reported inconsistent results. Pathophysiologically, preeclampsia is characterized by disturbances in oxidative, coagulative and vasomotor balance, partly due to increased sensitivity to angiotensin II, reduced production of vasodilatory prostaglandins, heightened sympathetic activity, hyperlipidemia with enhanced lipo-peroxide formation and incomplete cytotrophoblast invasion of the maternal spiral arteries. Elevated triglycerides in pregnancy-induced hypertension may deposit in vulnerable vessels, including uterine spiral arteries, contributing to endothelial dysfunction [9]. Abnormal lipid metabolism has also been strongly linked to atherosclerotic cardiovascular disease and directly affects endothelial integrity [10]. In normal pregnancy, the most pronounced lipid change is serum hypertriglyceridemia, which can increase two- to threefold by the third trimester compared with non-pregnant women. However, there is ongoing debate regarding total cholesterol changes in preeclampsia and findings across studies remain inconsistent [11, 12-13]. Due to these variations, generalizing lipid alterations to broader populations is challenging. Despite extensive research, the exact etiology of preeclampsia remains elusive and there is still no universally reliable clinical screening method to identify women at risk [14, 15]. Although numerous studies have explored the association between lipid abnormalities and preeclampsia, many reports conflicting results regarding the specific lipid parameters involved and their relationship with disease severity. Furthermore, most research has been conducted in non-Bangladeshi populations, limiting the applicability of findings to local maternal populations. Therefore, it is of interest to evaluate the impact of abnormal lipid profiles on the incidence and severity of preeclampsia.

## Methodology and Materials:

This retrospective cross-sectional study was conducted at the Department of Obstetrics & Gynecology, Bangabandhu Sheikh Mujib Medical University (BSMMU), Dhaka, Bangladesh, from March 2020 to February 2021. A total of 300 pregnant women were included, comprising 45 women diagnosed with preeclampsia and 255 normotensive controls, selected based on specific inclusion criteria.

## Inclusion criteria:

[1] Pregnant women attending the antenatal clinic at BSMMU during the study period.

[2] Women with available first-trimester or early-pregnancy lipid profile results.

[3] Women diagnosed with preeclampsia based on standard clinical criteria or normotensive controls.

## Exclusion criteria:

[1] Women with pre-existing chronic illnesses affecting lipid metabolism (*e.g.*, diabetes mellitus, hypothyroidism, hyperlipidemia).

[2] Women with multiple pregnancies (twins, triplets, *etc.*).

[3] Women with incomplete clinical or laboratory data.

[4] Women on lipid-lowering medications prior to or during pregnancy.

Demographic and clinical data-including maternal age, gestational age at enrollment, parity, pre-gestational body mass index (BMI) and family history of hypertension-were recorded for all participants. Fasting blood samples were collected to assess lipid profile parameters, including total cholesterol, triglycerides, high-density lipoprotein cholesterol (HDL-C) and low-density lipoprotein cholesterol (LDL-C). The primary outcomes were the incidence and severity of preeclampsia, while secondary outcomes evaluated the association of lipid abnormalities with disease occurrence and severity. Data were analyzed using SPSS software (version 25.0; IBM Corp., Armonk, NY, USA); continuous variables were expressed as mean ±standard deviation and compared using Student's t-test, while categorical variables were presented as frequency and percentage and compared using the Chi-square test. A p-value <0.05 was considered statistically significant.

## Results:

Among the 300 pregnant women included in the study, baseline characteristics showed no significant differences in maternal age (23.2±4.0 vs. 24.1±3.8 years; p = 0.165) or gestational age at enrollment (33.4±2.7 vs. 34.2±2.6 weeks; p = 0.071) between women with preeclampsia and normotensive controls. However, primiparity was more frequent in the preeclampsia group (55.6% vs. 35.3%; p = 0.014) and these women also had higher pre-gestational BMI (27.0±3.1 vs. 25.0±2.6 kg/m^2^; p < 0.001) and a greater family history of hypertension (40% vs. 21.6%; p = 0.011), suggesting these as potential risk factors (Table 1 - see PDF). The overall incidence of preeclampsia was 15% (n = 45), with 30 cases (10%) classified as mild and 15 cases (5%) as severe, while 255 women (85%) remained normotensive (Table 2 - see PDF). Analysis of lipid profiles revealed significantly higher mean levels of total cholesterol (242.7±61.2 vs. 195.8±34.1 mg/dL; p < 0.001), triglycerides (275.1±82.3 vs. 181.4±47.6 mg/dL; p < 0.001) and LDL-C (143.8±52.7 vs. 102.3±30.5 mg/dL; p < 0.001) among preeclamptic women compared to normotensive women, whereas HDL-C did not differ significantly (49.6±10.8 vs. 50.7±11.4 mg/dL; p = 0.534) (Table 3 - see PDF). Stratification by severity demonstrated that severe preeclampsia was associated with higher total cholesterol (277.3±68.9 vs. 225.4±50.1 mg/dL; p = 0.015), triglycerides (353.7±75.2 vs. 235.8±60.5 mg/dL; p < 0.0001) and LDL-C (170.4±60.1 vs. 130.5±40.3 mg/dL; p = 0.028) compared to mild disease, while HDL-C levels were similar (p = 0.640) (Table 4 - see PDF).

## Discussion:

Impact of abnormal lipid profiles on the incidence and severity of preeclampsia among pregnant women at a tertiary care hospital in Bangladesh. Preeclampsia, a pregnancy-specific disorder characterized by hypertension and multi-organ involvement, poses significant risks to both maternal and fetal health, often leading to adverse outcomes. The findings highlight the multifactorial nature of the condition, with factors such as primiparity, elevated pre-gestational BMI, positive family history of hypertension and dyslipidemia contributing to its development and progression. The observed association between abnormal lipid levels-particularly elevated total cholesterol, triglycerides and LDL-C-and both the occurrence and severity of preeclampsia underscores the potential value of early lipid assessment and targeted interventions to improve maternal and neonatal outcomes. In the present study, maternal age was comparable between preeclamptic and normotensive women (23.2±4.0 vs. 24.1±3.8 years; p = 0.165), which aligns with findings of Nahid *et al.* [16] (22.9±4.1 vs. 23.8±3.7, p = 0.169), Salma *et al.* [17] (23.09±2.1 vs. 24.71±2.56) indicating that maternal age may not significantly differ between groups. Gestational age at enrollment was slightly lower in the preeclampsia group (33.4±2.7 vs. 34.2±2.6 weeks; p = 0.071), similar to Nahid *et al.* [16], who reported non-significant differences, whereas Kondakasseril *et al.* [18] observed a slightly higher gestational age in their normotensive group, highlighting population variability. Primiparity was significantly higher in women with preeclampsia (55.6% vs. 35.3%; p = 0.014), consistent with the significant gravidity association reported by Nahid *et al.* [16] Pre-gestational BMI was higher in the preeclampsia group (27.0±3.1 vs. 25.0±2.6; p < 0.001), consistent with the findings of Nahid *et al.* [16], who reported 28.3±3.3 versus 26.1±2.4 (p = 0.019), highlighting maternal overweight as a significant risk factor. Additionally, a positive family history of hypertension was more frequent in preeclamptic women (40% vs. 21.6%; p = 0.011), paralleling the significant past history of hypertension reported by Nahid *et al.* [16]. Overall, these findings corroborate previous studies, emphasizing that parity, BMI and familial predisposition are important differentiating factors for preeclampsia, while age and gestational age show minor or non-significant differences.

In the present study, 15% of the 300 pregnant women developed preeclampsia, with 10% classified as mild and 5% as severe, while 85% remained normotensive. This distribution is consistent with Mou *et al.* [2], who reported a 14.4% prevalence of preeclampsia in Bangladesh, including approximately 10% mild cases and 85.6% normotensive participants. Parvin *et al.* [18] also reported a similar classification among 120 antenatal patients into mild, severe and normotensive groups, supporting the categorization of disease severity used in this study. Determining the proportion and severity of preeclampsia in this cohort offers a clear basis for evaluating how abnormal lipid profiles contribute to both its incidence and severity, thereby aligning with the central objective of this study. In the present study, women with preeclampsia demonstrated significantly higher levels of total cholesterol, triglycerides and LDL-C compared to their normotensive counterparts, while HDL-C levels showed no significant difference between the groups. Similar findings were reported by Shweta *et al.* [19], who observed significantly elevated total cholesterol, triglycerides, VLDL and LDL levels in preeclamptic women compared to normotensive pregnant women, whereas HDL levels were comparatively lower. These findings are also in close agreement with those reported by Akter *et al.* [20], who observed a similar pattern of elevated total cholesterol, triglycerides and LDL-C in preeclamptic women with no significant change in HDL-C. Salma *et al.* [17], however, noted a significant reduction in HDL-C alongside increased total cholesterol, triglycerides and LDL-C, suggesting population-specific variations in lipid alterations. Taken together, these studies, including our findings, support the notion that dyslipidemia characterized by hypercholesterolemia, hypertriglyceridemia and elevated LDL-C plays a contributory role in the pathophysiology of preeclampsia, though the behavior of HDL-C appears to vary across populations. In this study, women with severe preeclampsia exhibited significantly higher total cholesterol, triglycerides and LDL-C compared to those with mild preeclampsia, while HDL-C levels did not differ significantly. These findings align with Kumari *et al.* [21], who reported progressive increases in serum cholesterol, LDL and triglycerides from mild to severe preeclampsia, indicating that dyslipidemia intensifies with disease severity. These results demonstrate that abnormal lipid profiles are associated not only with the occurrence of preeclampsia but also with the progression to more severe forms, emphasizing the relevance of lipid monitoring in at-risk pregnancies.

## Conclusion:

Preeclampsia is among 15% of the participants, with higher rates of primiparity, elevated pre-gestational BMI and a positive family history of hypertension among affected women. Lipid profile analysis demonstrated significantly higher total cholesterol, triglycerides and LDL-C in preeclamptic compared to normotensive women, while HDL-C levels were similar. Moreover, severe preeclampsia was associated with further elevations in total cholesterol, triglycerides and LDL-C compared to mild cases. Thus, we show that abnormal lipid profiles are closely associated with both the incidence and severity of preeclampsia.
